# Core concepts of spatial prioritisation in systematic conservation planning

**DOI:** 10.1111/brv.12008

**Published:** 2012-12-22

**Authors:** Aija S Kukkala, Atte Moilanen

**Affiliations:** Department of Biosciences, University of HelsinkiPO Box 65, FIN-00014, Helsinki, Finland

**Keywords:** conceptual basis, conservation assessment, history of conservation planning, linguistic uncertainty, spatial conservation prioritisation, terminology

## Abstract

Systematic conservation planning (SCP) is a field of conservation biology concerned with delivering on-the-ground actions that achieve conservation goals. It describes a set of operational models that cover both design and implementation of conservation, with a strong focus on mobilising the collective action typically required to implement conservation. SCP, as it was originally described, was composed of six different stages: collection of data, identification of conservation goals, evaluation of the existing protected area network, design of expansions, implementation of conservation action, and long-term maintenance of biodiversity in the network. Since then, the operational model has been expanded into several different variants. Conservation actions applied inside SCP include establishment and expansion of reserve networks and allocation of habitat restoration and management.

Within the broader context of SCP, there is a fundamental biogeographic-economic analysis frequently called spatial conservation prioritisation or conservation assessment, which is used for identifying where important areas for biodiversity are and how conservation goals might be achieved efficiently. Here, we review the usage and meaning of the 12 biogeographic-economic core concepts of SCP: adequacy, complementarity, comprehensiveness, effectiveness, efficiency, flexibility, irreplaceability, replacement cost, representation, representativeness, threat, and vulnerability. Some of the concepts have clear definitions whereas others may have alternative and possibly conflicting definitions. With a comprehensive literature review literature, we elucidate the historical backgrounds of these concepts, the first definitions and usages, alternative later definitions, key applications, and prior reviews. This review reduces linguistic uncertainty in the application of SCP. Since SCP is a global activity with a multitude of different stakeholders involved, it is vital that those involved can speak the same language. Through these concepts, this review serves as a source of information about the historical development of SCP. It provides a comprehensive review for anyone wishing to understand the key concepts of spatial prioritisation within SCP.

## I. INTRODUCTION

**Fig. 1:**
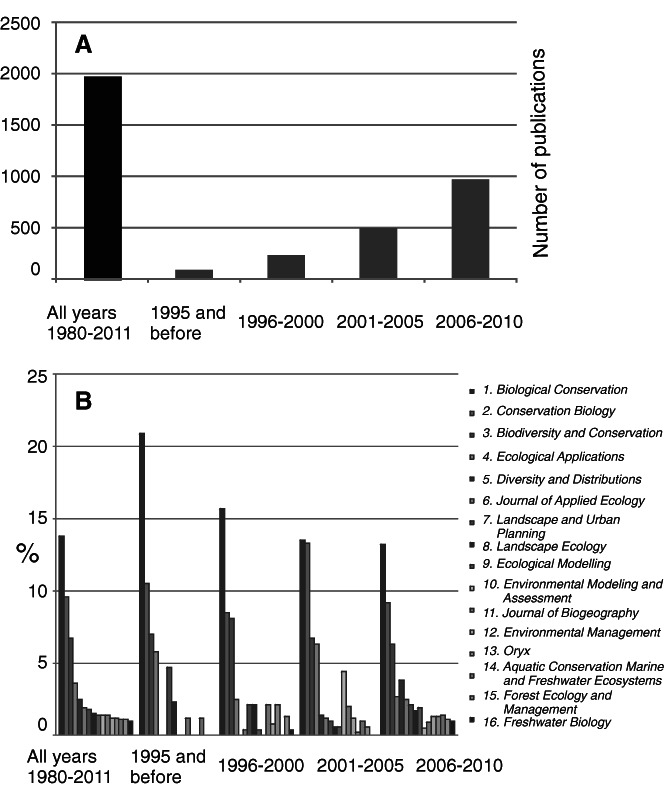
Publication volume and publication venue for our targeted search in systematic conservation planning (SCP), updated 23 September 2012. Statistics were divided into five intervals of publication years (all years 1980–2011, 1995 and before, 1996–2000, 2001–2005 and 2006–2010). (A) The total number of articles in our targeted search for SCP. (B) The percentage of articles appearing in each of the 16 top journals that have published the largest number of SCP publications; percentages for journals sum to 100%.

Systematic conservation planning (SCP) is widely considered as the most influential paradigm to identify and bring under protection priority areas for conservation (Margules & Pressey, 2000; Knight, Cowling & Campbell, 2006*a*; Pressey & Bottrill, 2009; Knight *et al*., 2010; Sarkar & Illoldi-Rangel, 2010). It concerns the prioritisation of sites for their biodiversity value and the participatory planning and collaborative implementation of strategies, decisions and actions that secure the long-term survival and favourable conservation status of biodiversity in general. In the early 1990s it was observed that biodiversity was being lost and that the development of reserve networks had mostly occurred in an *ad hoc* manner, with areas protected due to their special location or aesthetic values (Pressey *et al*., 1993). SCP was developed to tackle the biodiversity crisis and to address this bias in existing conservation areas. The fundamental conceptual basis and process of SCP can be dated to that time; its original operational model was influentially summarised by Margules & Pressey (2000). Environmental philosophers have described SCP as the first ‘consensus view’ in conservation biology (Sarkar, 2005). Publications on SCP and related fields have increased in volume almost exponentially during the past 20 years, with the disciplines maturing and broadening in scope (Fig. 1).

At the outset we should clarify terminology and the scope of this study. SCP focuses on delivering actions that achieve conservation goals, which involves a significant socio-political component (e.g. Knight *et al*., 2006*a*, 2010; Margules & Sarkar, 2007). Within SCP there is a fundamental biogeographic-economic activity of identifying important areas for biodiversity; where, when and how we might efficiently achieve conservation goals (Pressey *et al*., 2007; Wilson *et al*., 2007; Ferrier & Wintle, 2009). This activity can be called spatial conservation prioritisation and is our present focus. It also is closely synonymous to conservation assessment, i.e. quantitative assessment of conservation value and potential (Fuller *et al*., 1991; Noss *et al*., 2002; Knight *et al*., 2006*a*; Ferrier & Wintle, 2009). Herein we concentrate on the core concepts of SCP relevant for the setting of conservation objectives and biogeographically and economically informed solution of spatial prioritisation problems.

SCP is a multidisciplinary science; it employs methodology from many other fields of science such as spatial ecology, sociology, geography, computer sciences, mathematics, and economics (Lindenmayer & Hunter, 2010; Reyers *et al*., 2010). It is also a discipline of decisions, with spatial analyses providing decision support for real-world decision-making (Harwood, 2000; Pullin *et al*., 2004; Wilson *et al*., 2006). So-called reserve selection (also known as site selection, area selection, reserve design, or reserve network design) is a specific kind of resource allocation problem that is frequently encountered in spatial prioritisation. Linking to applied mathematics, reserve selection can be formulated as a classic optimisation problem with an objective function, constraints and a mathematical description of our knowledge of the system (e.g. Pressey, Possingham & Day, 1997; Margules, Pressey & Williams, 2002; Williams, ReVelle & Levin, 2004; Sarkar *et al*., 2006; Moilanen, Possingham & Polasky, 2009*c*). SCP must deal with conservation challenges in an uncertain world (Harwood, 2000; Meir, Andelman & Possingham, 2004; Burgman, Lindenmayer & Elith, 2005; McCarthy *et al*., 2011), often in a situation where there are not enough data or data are sparse and incomplete (Polasky *et al*., 2000; Gaston & Rodrigues, 2003). As conservation competes with other land uses in the real-world, many studies have investigated how socio-economic and political factors affect conservation solutions (Naidoo *et al*., 2006; Wilson *et al*., 2007; Nelson *et al*., 2009; Adams, Pressey & Naidoo, 2010). A stronger socio-political emphasis in SCP has brought attention to stakeholder collaborations, social learning, and links with general land-use planning (Knight *et al*., 2006*a*, 2010). All these components bring special characteristics, analyses, and terminology into SCP, which does not necessarily facilitate easy uptake of literature and methods for anyone new to the broad discipline.

SCP is a stage-wise operational model for the planning and implementation of conservation (Knight *et al*., 2006*b*; Margules & Sarkar, 2007; Sarkar & Illoldi-Rangel, 2010), and was originally described as consisting of six stages (Margules & Pressey, 2000). Thereafter, the applicability of the original model was improved in several studies that discussed the limitations and developed different expanded variants of the original work (Cowling & Pressey, 2003; Knight *et al*., 2006*a*, *b*, 2011*a*; Conservation Measures Partnership, 2007; Margules & Sarkar, 2007; Pressey & Bottrill, 2009; Sarkar & Illoldi-Rangel, 2010). The operational model of SCP was thus expanded to 10 (Sarkar, 2005), 11 (Pressey & Bottrill, 2009) or 13 stages (Sarkar & Illoldi-Rangel, 2010). Discussion around the SCP model has mostly concentrated on the interactions among components and on revision and reiteration of planning stages due to feedbacks among them (Sarkar & Illoldi-Rangel, 2010).

Following the stages in Pressey & Bottrill (2009) as an example, the first stage is to delimit the planning area (Pressey & Bottrill, 2009; Sarkar & Illoldi-Rangel, 2010). The second and the third stages are to identify all stakeholders and describe the context for conservation areas. Cowling & Pressey (2003) introduced the idea that identification of stakeholders should be considered as a distinct component of SCP. The fourth stage of SCP concerns the identification of broad conservation goals, for example about representation, persistence, ecosystem services, and livelihoods.

The fifth and sixth stages of SCP are collection of data across the focal landscape (Pressey & Bottrill, 2009). Information is needed about the distributions of various classes of biodiversity features, which may include species, habitat types, ecosystem services, ecosystem processes, genes, etc. Other data relevant to SCP include socio-economic variables and threats, information about land cost, opportunity costs for stakeholders, and various information about anthropogenic influences that might influence land use and landscape structure in the future. The seventh stage concerns setting of targets (quantitative conservation objectives) for biodiversity features. Conceptually, targets are often based on the principle of adequacy, which specifies, for example, that the extinction risk of a species must be low or the conservation outcome is not adequate. The eighth stage of SCP concerns evaluation of the existing protected area network, i.e. assesses current achievement of previously developed objectives. At this stage the technique of gap analysis is frequently used, to identify deficiencies in the conservation coverage of biodiversity (Scott *et al*., 1993; Kiester *et al*., 1996; Rodrigues *et al*., 2004*a*).

The ninth stage of SCP fundamentally concerns the biogeographical activity of spatial conservation prioritisation or conservation assessment. It requires identifying important areas for protected area network expansion or management (Pressey & Bottrill, 2009). In this stage, decision-theoretic methods from the field of applied mathematics are frequently applied. So-called reserve selection or site selection algorithms are optimisation methods that are used to identify the ‘best possible’ reserve network (Csuti *et al*., 1997; Pressey *et al*., 1997). Conservation planning software such as Marxan (Ball & Possingham, 2000) and ConsNet (Ciarleglio, Barnes & Sarkar, 2009) implement optimisation algorithms while C-Plan (Pressey *et al*., 2009) implements so-called irreplaceability analysis.

The 10th stage of SCP deals with the implementation of conservation action, in which socio-economic and political considerations are accounted for together with ecological considerations (Knight *et al*., 2006*a*; Wright *et al*., 2007). Finally, for conservation to be successful, biodiversity should be maintained in the reserve network and across the landscape. This may require habitat management, maintenance, and restoration actions (Hobbs & Norton, 1996; Dobson, Bradshaw & Baker, 1997; Crossman & Bryan, 2006). Maintaining and monitoring of conservation areas is the 11th stage of SCP. The most frequent application of SCP is a one-off project, but various stages of SCP may be repeated, for example, following major changes in information or available resources (Brooks *et al*., 2006; Knight *et al*., 2006*b*; Ferrier & Wintle, 2009).

SCP frequently involves identifying a reserve network that best satisfies a number of principles: comprehensiveness, representativeness, adequacy, efficiency, flexibility, and irreplaceability (Possingham *et al*., 2006). Frequently cited key concepts also include complementarity, which concerns selection of complementary areas to avoid duplication of effort, and threat and vulnerability, which concern the persistence of biodiversity features in focal areas (Margules & Pressey, 2000; Cabeza & Moilanen, 2001; Sarkar *et al*., 2006; Moilanen, 2008). Complementarity is often cited as the defining concept of SCP (Williams, 2001; Funk & Richardson, 2002; Justus & Sarkar, 2002; Margules & Sarkar, 2007; Moilanen, 2008). These concepts are the focus of the present work. We emphasise that this focus is principally on the philosophy of SCP rather than its application. We concentrate on the oldest SCP concepts that concern the definition of conservation objectives and the solution of reserve selection problems in spatial conservation prioritisation and conservation assessment. For reviews on the socio-political aspects of SCP and about implementation, mainstreaming, and enabling of conservation action, we refer the reader to Knight *et al*. (2006*a*), Margules & Sarkar (2007) and Knight *et al*. (2010).

The importance of clearly describing the process of SCP has been noted and summarised by several authors (e.g. Knight *et al*., 2006*a*, *b*; Regan *et al*., 2007; Pressey & Bottrill, 2009; Sarkar & Illoldi-Rangel, 2010). However, since the early development of SCP, only a few studies have focused on its concepts and terminology (Pressey *et al*., 1993; Justus & Sarkar, 2002; Possingham *et al*., 2006; Sarkar *et al*., 2006; Margules & Sarkar, 2007; Moilanen, 2008; Wilson, Cabeza & Klein, 2009). Linke, Turak & Nel (2011) evaluated key principles of conservation, primarily from the point of freshwater conservation. These prior studies each have their own focus, but none provides a full up-to-date review of the core concepts of SCP. Presently, threats to biodiversity remain. Habitat loss is continuing in many countries (Cowling *et al*., 2003; Fahrig, 2003; Polasky *et al*., 2005) and global warming appears to be progressing rapidly (Araujo *et al*., 2004; Parmesan, 2006; UNEP, 2011). Following the resolution by the Convention on Biological Diversity to almost double the extent of the world's protected areas by 2020 (Normile, 2010; UNEP/CBD, 2010), there will be widespread demand for methods and operational models by which conservation resources can be allocated spatially in an effective manner. Here, we undertake a comprehensive review of the core concepts of spatial prioritisation within SCP, reducing linguistic uncertainty around these concepts, and supporting urgent global conservation efforts by improving the accessibility of this major field of conservation biology.

## II. METHODS

We selected 12 main SCP terms to focus on: adequacy, comprehensiveness, representativeness, representation, complementarity, threat, vulnerability, effectiveness, efficiency, irreplaceability, flexibility, and replacement cost. These are all concepts that display prominently in the literature on SCP and spatial prioritisation, but which may have variable definitions. We started our review by collating comprehensive literature about SCP, spatial conservation prioritisation, spatial optimisation, and other topics connected to spatial allocation of conservation effort. We searched for citations from Thomson Reuters' *Web of Science* by using the search: topic = [‘reserve network’ OR (‘reserve design’ AND conservation) OR ‘reserve selection’ OR ‘site selection algorithm’ OR ((systematic OR spatial OR quantitative OR metapopulation) AND ‘conservation planning’) OR ‘reserve site selection’ OR ‘connectivity conservation’ OR (conservation AND (‘spatial optimization’ OR ‘spatial optimisation’)) OR ‘conservation prioritisation’ OR ‘conservation area prioritisation’ OR ‘conservation area selection’ OR ‘protected area network’ OR ‘conservation resource allocation’ OR ‘conservation decision analysis’]. Search results were downloaded from the *Web of Science*, and PDFs of publications were retrieved within the limits of the library access permissions of the University of Helsinki. We were able to access approximately 90% of the 1834 publications found by this targeted search, providing us with a database of 1659 PDFs, covering years from 1980 to June, 2011. These publications consisted mostly of primary research papers, but also of reviews, opinion pieces, and, to a lesser extent, book chapters and conference papers. Upon inspection, it was discovered that all except one of these studies were relevant to spatial conservation in the broad sense, demonstrating a good specificity of the search phrase.

The targeted search was used as a starting point for our investigation. A full text search was applied using the Adobe Acrobat X and PowerGREP tools (Powergrep, 2011), and every mention of the core terms inside these 1659 PDFs was investigated. Thus, we can be confident that a fairly broad literature base was covered thoroughly. Literature chains were followed outside the basic set when appropriate. Older articles and books not available in electronic text form were investigated manually. Known prior summaries into SCP or spatial conservation prioritisation were also included as starting points (Margules & Pressey, 2000; Justus & Sarkar, 2002; Sarkar *et al*., 2006; Margules & Sarkar, 2007; Moilanen, 2008; Moilanen, Wilson & Possingham, 2009*d*; Linke *et al*., 2011).

We investigated each term by reviewing its historical heritage, when the term was first mentioned, how it has been defined and discussed, and how definitions and usage have changed through time. This review contains verbatim quotes from original key publications to avoid mistakes in interpretation about the original definition.

We have divided our timeline into three phases: (*i*) the historical period; (*ii*) the classical period; and (*iii*) the period of expansion. By the historical period we mean the period from the 1950s to the end of the 1980s when the ideas underlying SCP concepts were forming but the present concepts had not yet been explicitly named and defined. By our definition, the classical period (1990s) was when most SCP concepts became firmly named and their early definitions were discussed. The period from 2000 to the present is described as a period of expansion, reflecting consolidation of the field, a rapidly increasing publication count, and a tendency of methods to become more inclusive of new analysis features. This period begins from the influential work of Margules & Pressey (2000), who formulated an early operational model of SCP, successfully bringing wide recognition to the field.

## III. RESULTS

### (1) Occurrence of terms in the literature

**Fig. 2:**
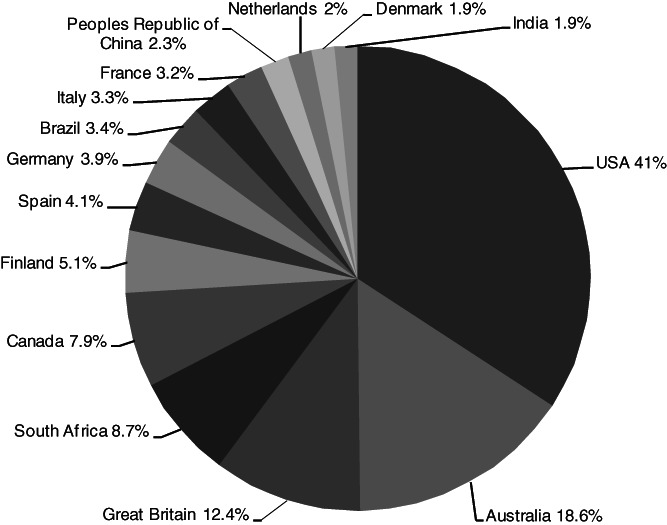
Country of origin of publications in systematic conservation planning and spatial conservation during 1980–2011. The percentages for countries do not sum to 100%, as they indicate the fraction of studies that include at least one author from the country in question; many publications include authors from several countries. In total, authors were present from 104 different countries.

Figures 1 and 2 summarise publication volume across years, publication venues, and countries of origin of research. Most of the literature in the field of SCP and spatial conservation prioritisation in the broad sense is relatively recent – over 50% of the articles were from 2006 or later. The count of new publications in the field has approximately doubled each 5-year period since 1995 (Fig. 1A). The majority (95%) of studies were published in scientific journals consisting of original research articles and reviews, the remaining 5% were from book chapters and conference proceedings, reflecting the practice in ecology and conservation biology to publish primarily in scientific journals. The count of journals publishing this work was 37 up to the end of 1995, increasing to 273 during 2006–2010. The top five journals in this field (*Biological Conservation*, *Conservation Biology*, *Biodiversity and Conservation*, *Ecological Applications*, and *Diversity and Distributions*) jointly accounted for 36% of the total publications since 1980 (Fig. 1B). The country of origin of published work was strongly biased towards the United States of America, Australia, and Great Britain (Fig. 2); other countries such as Australia, South Africa, and Finland were overrepresented relative to their population size.

Table 1 provides information about the occurrence of concepts and terms of SCP in scientific literature. The frequency of use for almost all terms increases with time, primarily reflecting the increasing amount of literature available. Several of the 12 key concepts occurred frequently (in over 25% of publications), reflecting the prevalence of the basic concept set (Margules & Pressey, 2000; Justus & Sarkar, 2002; Moilanen, 2008; Wilson *et al*., 2009). These terms included complementarity, effectiveness, efficiency, representation, and threat (Table 1). Somewhat less discussed key concepts (present in 5–20% of publications) included adequacy, flexibility, irreplaceability, representativeness, and vulnerability. Note that many of these terms are common English words that nevertheless have special connotations in the context of SCP. Of supplementary terms target, goal, and objective occurred very frequently (in approximately 40–60% of all publications), implying that the target-oriented model of specifying objectives and trying to satisfy them efficiently has been widely adopted (Nicholson & Possingham, 2006; Carwardine *et al*., 2009; Moilanen & Arponen, 2011). The statistics provided in Table 1 can be used to provide an overview of the use of terminology in SCP, for identifying research trends, and for identifying informative key words for literature searches. Knowing the frequency of the use of a term may help in the planning of literature searches; a very frequently occurring term is on its own too unspecific while a very infrequently occurring phrase may fail sufficiently to identify relevant literature. We also found that success in searches for terms was highly variable from article titles and abstracts or from inside the article text: some terms occur predominantly in the body text of an article and can thus only be located using a full-text PDF search (Table 1).

**Table 1:** Occurrences of terms and concepts of systematic conservation planning and spatial conservation in scientific literature

	WOS broad conservation search (*N* = 75697)	Targeted search (*N* = 1834), June 2011									
		
		Hits in titles and abstracts							Hits inside full-text PDF files (*N* = 1659)		
				
	Number of publications (hits in abstracts, key words and titles)	Titles and abstracts (all years)	Only titles	Before 1995 (*N* = 96)	1996–2000 (*N* = 235)	2001–2005 (*N* = 485)	2006–2010 (*N* = 936)	Hits in key words	Hits	Files	Files %
*Statistics for 12 key concepts of systematic conservation planning* (*SCP*)											
Adequacy	103	24	3	0	4	3	12	5	270	138	8.3
Complementarity	348	216	20	3	32	88	84	139	2537	426	25.7
Comprehensiveness	9	8	0	0	0	4	3	0	56	40	2.4
Effectiveness	1499	261	43	1	20	41	182	15	2129	547	33.0
Efficiency	1703	210	20	4	41	54	100	0	2307	464	28.0
Flexibility	385	34	1	2	9	5	17	0	607	290	17.5
Irreplaceability	58	120	8	6	12	38	58	97	2744	228	13.7
Replacement cost	2	10	2	0	0	0	9	1	141	10	0.6
Representation	1039	468	27	10	68	133	232	56	6633	834	50.3
Representativeness	90	63	8	6	9	22	24	29	603	178	10.7
Threat	3491	298	19	0	27	66	178	33	4415	1036	62.4
Vulnerability	913	96	11	1	13	43	37	53	1650	296	17.8
*Statistics for supporting concepts and terms*											
Area selection	41	43	4	0	13	15	12	35	536	156	9.4
Benefit function	5	8	1	0	0	0	8	4	157	27	1.6
Biodiversity	17889	2011	375	35	234	567	1067	1011	17216	1280	77.2
Biodiversity feature	27	45	0	0	2	15	20	1	534	142	8.6
Climate change	3548	242	39	0	2	46	165	110	2243	340	20.5
Community	9490	292	18	25	33	44	179	106	3966	693	41.8
Connectivity	2615	376	39	2	13	44	265	156	4884	519	31.3
Conservation assessment	75	27	7	0	2	3	22	11	301	82	4.9
Conservation prioritisation	60	73	20	0	4	14	50	27	366	125	7.5
Cost	2960	647	40	6	59	138	373	95	12014	1041	62.7
Cost-effectiveness	123	22	3	0	1	3	10	7	203	60	3.6
Decision analysis	74	6	0	0	0	3	2	3	95	51	3.1
Dispersal	6856	280	23	4	15	87	159	125	5651	604	36.4
Distinctiveness	194	23	2	0	4	2	14	2	272	65	3.9
Ecosystem service	910	97	12	0	0	11	83	26	959	161	9.7
Ecosystem	12134	614	84	10	52	130	388	164	7320	987	59.5
Endangered species	2353	83	10	3	17	28	27	33	995	358	21.6
Endemic species	718	66	3	2	10	19	34	8	1345	290	17.5
Endemism	686	157	18	7	33	37	70	66	2014	352	21.2
Evolution	6494	44	20	3	3	12	26	16	670	279	16.8
extinction	5820	170	10	9	30	30	98	159	3533	617	37.2
Extinction risk	869	29	2	3	4	4	18	35	559	125	7.5
Feature	4601	242	3	12	32	53	118	2	6118	961	57.9
Flagship species	99	5	1	0	2	1	2	1	122	53	3.2
Fragmentation	6409	197	18	13	24	63	84	174	2233	541	32.6
Genetic	13185	272	32	6	28	63	164	52	3504	421	25.4
Goal	2615	277	7	9	34	89	140	12	4785	971	58.5
Habitat loss	1376	103	7	1	19	28	54	44	1019	340	20.5
Habitat management	456	10	0	0	0	5	5	0	68	45	2.7
Habitat type	461	82	2	0	12	14	48	0	1973	493	29.7
Hotspot	1509	377	45	6	64	131	170	238	4377	428	25.8
Indicator species	360	27	2	2	8	8	9	14	407	119	7.2
Invasive species	994	15	3	0	0	0	11	4	292	137	8.3
Isolation	3348	66	1	1	11	27	25	12	1057	340	20.5
Keystone species	191	4	0	1	1	0	2	2	65	45	2.7
Metapopulation	2292	120	11	6	13	35	53	101	1704	266	16.0
Objective	3950	329	14	3	31	111	169	15	5799	686	41.4
Opportunity cost	92	62	6	0	4	11	37	10	1083	158	9.5
Optimality	56	15	4	0	4	5	5	9	277	107	6.4
Optimisation	779	146	19	1	23	39	79	135	2026	368	22.2
Persistence	2792	286	24	3	40	80	134	101	3808	668	40.3
Protected area network	49	128	24	5	16	21	73	36	601	167	10.1
Reserve design	301	311	60	32	48	92	128	217	2091	461	27.8
Reserve selection	312	385	74	26	66	120	162	513	2943	550	33.2
Reserve selection algorithm	59	74	9	9	19	20	26	83	518	190	11.5
Restoration	3506	153	19	2	6	17	112	39	1996	333	20.1
Retention	726	44	5	0	4	6	32	9	810	153	9.2
Scoring	93	26	1	6	3	10	7	2	637	193	11.6
Site selection	583	142	32	9	16	56	58	167	1707	361	21.8
Species	38773	5848	289	212	696	1539	3074	704	103420	1552	93.6
Stakeholder	668	94	2	0	0	18	69	4	1387	231	13.9
Surrogate	358	201	20	0	20	44	135	37	2602	471	28.4
Sustainability	1798	41	5	1	4	16	17	23	381	172	10.4
Systematic conservation planning	102	150	20	0	1	24	102	83	868	297	17.9
Target	2261	613	37	16	43	163	328	53	12754	1050	63.3
Umbrella species	79	27	3	0	1	10	14	8	336	78	4.7

The first section of the table gives statistics for the 12 key concepts and the second section for a number of supplementary terms and concepts. The first column gives the search term and the second column gives the number of hits in the ISI *Web-of-Science* search of a broad set of literature covering conservation biology in general, approximately 75000 publications [topic = (biodiversity OR conservation OR landscape) AND (population OR metapopulation OR ecology OR spatial)]. The next seven columns summarise occurrences in the titles, abstracts and key words of the targeted SCP search (*N* = 1834 articles, described in Section II). The last three columns summarise occurrences inside full-text PDFs of the targeted search, excluding literature sections of publications.

### (2) Adequacy

Adequacy already was seen as an important concept in reserve design and nature conservation by the 1990s. It can be defined as ‘*the maintenance of the ecological viability and integrity of populations*, *species and communities*’ (Commonwealth of Australia, 1992, glossary, *iii*) or ‘*as the extent to which reserves fulfil their basic purpose of conserving biodiversity*.’ (Lunney *et al*., 1997, p. 138).

Persistence is frequently mentioned as underlying adequacy (Cowling & Pressey, 2001; Pressey & Logan, 1998; Williams & Araujo, 2000; Desmet & Cowling, 2004; Wilson *et al*., 2009; Linke *et al*., 2011). The idea of adequacy and species persistence goes back to studies of the species-area relationship, colonisation and extinction theories, and island biogeography theory from the 1960–1970s. A cornerstone of this research is MacArthur & Wilson's (1963) study on immigration and extinction curves. Island biogeography theory influenced conservation *via* discussions about how the size and other features of conservation areas (or ‘islands’) influence the persistence of species (Diamond, 1975; Simberloff & Abele, 1976; Margules, Higgs & Rafe, 1982). During the 1970–1980s the importance of area size and shape, extinction and colonisation rates, and species-area relationships were much discussed from the perspective of how they should influence the design of adequate single reserves or reserve systems (Margules & Usher, 1981; Saunders, Hobbs & Margules, 1991). The first use of the term adequacy is difficult to identify; the concept was probably proposed multiple times in different contexts, both in the scientific literature and agency documents. Early definitions for adequacy were given by Soulé (1986), Margules & Stein (1989) and Nicholls & Margules (1993), with key principles applied in conservation planning already by the early 1990s. Adequacy was one of the ‘CAR concepts’ (comprehensiveness, adequacy, and representativeness) established in the context of Australian forest reserves (Commonwealth of Australia, 1992; JANIS, 1997; Linke *et al*., 2011).

Adequacy gained attention in relation to target-based conservation planning (Pressey *et al*., 2000; Williams & Araujo, 2000; Klein *et al*., 2009). As Wilson *et al*. (2009, p. 19) define the concept: ‘*One approach to address adequacy in spatial conservation prioritization is to set conservation goals in the form of a target percentage of original extent or a target population size that is large enough to ensure persistence of the feature*.’ This definition followed from possibly the first quantitative approach to target-setting to ensure persistence by Desmet & Cowling (2004). Crossman & Bryan (2006, p. 372) see adequacy in more general terms: ‘*That is*, *increasing patch size and connectivity to improve the chances that species will be able to maintain viable populations*.’ Possingham *et al*. (2005) determine adequacy of a reserve system by assessing the viability of key species. Ecological and evolutionary processes that support persistence of biodiversity should be accounted for when addressing adequacy (Cowling *et al*., 1999, 2003; Klein *et al*., 2009; Linke *et al*., 2011).

As a criticism to considerations of conservation area size, Margules & Sarkar (2007) state that adequacy cannot be measured in terms of area of land. Instead, it depends on the extent to which conservation area networks sample the range of natural variation and on whether biotic features there are likely to persist into the future. This use of adequacy is in fact close to earlier usages of comprehensiveness (Section III.3). In particular, representation of biodiversity features is not the same as long-term persistence (Pressey & Logan, 1998). According to Possingham *et al*. (2005), inadequate understanding of landscape heterogeneity is perhaps one reason why adequacy has not been accommodated very well in conservation.

In summary, adequacy remains a complicated concept without a precise definition (Saunders *et al*., 1991; Powell, Barborak & Rodriguez, 2000). Wilson *et al*. (2009) acknowledge that lack of data and uncertainty complicate and reduce research about persistence and adequacy. According to Linke *et al*. (2011), long-term commitment in ecological research is needed when addressing how adequacy and persistence and distributions of species depend on habitat dynamics and ecological processes.

### (3) Comprehensiveness

Comprehensiveness is not the most commonly used SCP concept (Table 1) and it is frequently encountered together with the other two CAR terms, adequacy and representativeness. In the 1990s, when the SCP paradigm was developing, the term comprehensiveness was first used in the context of goal-setting of spatial prioritisation in Australia (Commonwealth of Australia, 1992; JANIS, 1997; Linke *et al*., 2011). The Commonwealth of Australia's (1992, glossary, *iii*) definition is: ‘*Comprehensiveness includes the full range of forest communities recognised by an agreed national scientific classification at appropriate hierarchical levels*.’ Later, Possingham *et al*.'s (2006, p. 520) definition for the concept of comprehensiveness underlines the importance of and challenges in achieving conservation area quality and broad coverage of biodiversity: ‘*A comprehensive reserve system is one that contains examples of many biodiversity features*, *where biodiversity features might include species*, *habitats, or ecological processes. While ideally we would like to include a sample of every kind of biodiversity feature in our reserve system this is rarely achieved*.’ We note that comprehensiveness is about conservation objectives and about defining the full spectrum of biodiversity. Comprehensiveness, as defined by both the JANIS (1997) criteria and Possingham *et al*. (2006) overlaps with the term representativeness (Section III.4).

Although the concept of comprehensiveness encompasses a certain idealism, there have been serious efforts to define a comprehensive reserve system. In early uses of this term, Franklin *et al*. (1981) and Franklin (1988, 1993) discuss compositional, functional, and structural diversity. At that time, most management strategies did not take biological diversity into consideration, and Franklin's research on species, habitats, and ecological processes can be considered one of the first efforts to incorporate biodiversity objectives into conservation management: ‘*Habitat reserves are an essential element in any comprehensive program to conserve biological diversity for the foreseeable future*. *The objective in designing a reserve system is to try to ensure that the reserves are sufficient in number and size and appropriately distributed over the landscape in terms of geography and ecosystem type*.’ (Franklin, 1993, p. 203). (Note the use of sufficiency here is close to the sense of adequacy.) Later, Noss (1990, p. 356) has sought a definition for comprehensive coverage of biodiversity: ‘*A definition of biodiversity that is altogether simple*, *comprehensive*, *and fully operational* (*i*.*e*., *responsive to real-life management and regulatory questions*) *is unlikely to be found*. *More useful than a definition*, *perhaps*, *would be a characterization of biodiversity that identifies the major components at several levels of organization*.’ Franklin *et al*.'s (1981) considerations on composition, structure, and function were still in use when Wilson *et al*. (2009, p. 16) defined the concept: ‘*In simple terms*, *a comprehensive network of priority areas is one that includes a portion of every biodiversity feature*. *More broadly the notion of comprehensiveness in conservation prioritization implies sampling the full range of biodiversity taking into account composition* (*e*.*g*. *species and genetic diversity*), *structure* (*e*.*g*. *habitat types*), *and function* (*e*.*g*. *recruitment and dispersal processes*).’ (Note that this definition includes elements of representativeness, see Section III.4). The idea of comprehensiveness can also be extended to the non-living environment (Noss, 1990; Bonn & Gaston, 2005).

The definition of comprehensiveness has not changed much from the 1990s – it can still be defined as the ‘as many species as possible’ principle (Burgman & Lindenmayer, 1998; Stewart, Noyce & Possingham, 2003; Beger & Possingham, 2008). However, comprehensiveness and representativeness have become more clearly defined and separated with respect to each other. Limitations to the application of the concept also remain (Wilson *et al*., 2009, p. 17): ‘*A fully comprehensive network of priority areas is not technically possible because spatial data on all aspects of biodiversity are not available for any region*’. Therefore, comprehensiveness can be achieved for a limited set of features, in a specific landscape, and at a limited spatial resolution – not for all features, everywhere, and at high resolution.

### (4) Representativeness

Representativeness is a relatively infrequently cited yet important SCP concept (Table 1). Margules & Pressey (2000, p. 243) described it as ‘*a long-established goal referring to the need for reserves to represent*, *or sample*, *the full variety of biodiversity*, *ideally at all levels of organization*.’ Margules & Sarkar (2007, p. 111) summarise: ‘*Representativeness can then be viewed as measured by the fraction of surrogates that meet their set targets*.’ Ferrier & Wintle's (2009, p. 12) alternative was: ‘*A basic measure of representativeness is simply the number of surrogate features represented* (*included*) *at least once somewhere in a reserve network*.’ As a distinction from comprehensiveness, we note that representativeness considers the properties of a solution (conservation area network) and how well it covers the biodiversity of the region. Representativeness was considered a fundamental selection criterion of nature reserves by the 1970s and 1980s (Wright, 1977; Austin & Miller, 1978; see Margules & Usher, 1981 for review; Austin & Margules, 1986). The problem of how to measure representativeness is central to SCP and much discussed in the context of the target-based planning approach (Margules & Pressey, 2000, p. 246): ‘*representativeness and persistence – have to be translated into more specific*, *preferably quantitative*, *targets for operational use*. *Targets allow clear identification of the contributions of existing reserves to regional goals and provide the means for measuring the conservation value of different areas during the area selection process*.’ Species richness or habitat diversity is usually related to representativeness (Austin & Margules, 1986; Newburn *et al*., 2005) and methods of operations research are often applied to optimise representation of a set of target taxa or habitat types (Bedward, Pressey & Keith, 1992; Margules & Sarkar, 2007; Ferrier & Wintle, 2009).

In the 1980–1990s, representativeness became embedded into conservation policies and conservation programmes, with several early developments published in Australia (e.g. Austin & Margules, 1984; Commonwealth of Australia, 1992; Belbin, 1993, 1995; Awimbo, Norton & Overmars, 1996; JANIS, 1997). The concept of typicalness was seen as a part of representativeness (Margules & Usher, 1981, p. 100): ‘*Areas selected to be representative would necessarily include typical or common species but they could also include rare species since their objective is to represent the range of biota*.’ Smith & Theberge (1986, p. 724) expanded this definition in their review: ‘*There are two differing definitions of representativeness*, *which we will call inclusive and typicalness*. *The definition from* Margules & Usher (1981) *is inclusive*. *This approach views the selection of reserves as a means to represent the full range of natural features in a system of reserves*. *A different view equates representativeness with typicalness*: *Representativeness and uniqueness can be the extremes of a spectrum*. *A* “*unique*” *area is one that is rare*, *whereas areas which are representative*…*are typical of a biome or habitat types*…’. It is obvious that representativeness could be understood in at least two very different ways. Later, Heink (2009, p. 322) saw representativeness as a combination of distinctiveness, typicalness, and comprehensiveness: ‘*We can distinguish three different meanings of representativeness*. *First*, *it can be seen as a measure of the characteristic inventory of species and habitats in geographic regions* (*distinctiveness*). *Second*, *representativeness is the degree to which a habitat conforms to a habitat type or to which the presence of a species is correlated with a habitat type* (*typicalness*). *Third*, *representativeness can be seen as the extent to which required natural features occur within a habitat or a* (*set of*) *site*(*s*) (*comprehensiveness*).’

Smith & Theberge (1986, p. 724) linked representativeness also to gamma diversity (Whittaker, 1972), which can be considered as an early linkage to the concept of complementarity (Section III.6): ‘*However, the idea of representativeness, particularly the* “*inclusive*” *definition, is related to gamma diversity. Classifications of natural diversity that are used in assessing representativeness, such as that of Radford and others (1981) are attempts to provide a framework for conserving a region's gamma diversity*.' A few years later Mackey *et al*. (1989, p. 281) brought the concept of representativeness into the context of geographic scale: ‘*As biotic variation can be examined at a range of resolutions, so can the environmental relations*. *Representativeness then must be assessed in the context of a geographic scale*. *Thus a property can be representative of the biophysical characteristics found within the surrounding locality, region, continent or globe*.’

Pressey & Taffs (2001) and Pressey *et al*. (2002) require other measures in addition to the concept of representativeness and state that it should be linked to the concept of efficiency: ‘*One shortcoming is that representativeness does not indicate whether new reserves add unnecessarily to the protection of features that are already represented to target levels*. *This problem is addressed here by a fourth measure – efficiency –which also reflects bias in conservation action since low efficiency will result from repeated*, *above-target reservation of some features at the expense of others that remain inadequately protected*. *Another shortcoming of representativeness is that it conveys no information on the relative need for protection of the features in a reserve system*.’ (Pressey *et al*., 2002, p. 58). This quote also includes clear references to adequacy and complementarity. Overall, representativeness is one of the most defined and discussed concepts in SCP (Stokland, 1997; Pressey & Taffs, 2001; Pressey *et al*., 2002; Kati *et al*., 2004; Sarkar *et al*., 2006). Nevertheless, from the varied nature of comments about representativeness, it seems apparent that the definition of the concept remains unclear. We will discuss the definitions of adequacy, comprehensiveness, and representativeness in Section IV.

### (5) Representation

Williams (2001, p. 813) defines the concept of representation as: ‘*The occurrence of species* (*or other attributes*) *within a set of selected areas*.’ Considering population sizes and ranges of species in reserve networks is an old idea in conservation biology (Wright, 1977; Margules & Usher, 1981; Margules, Nicholls & Pressey, 1988). However, measuring the level of representation – i.e. the exact amount of a feature in an area – is a complicated task, and importantly, may depend on the scale and resolution of measurement (Olson, 1998; Pressey & Logan, 1998; Sarkar *et al*., 2006).

In reserve selection, the concept of representation naturally leads to the concept of representation goal or target (Nilsson & Götmark, 1992; Margules & Pressey, 2000; Williams, 2001; Arponen *et al*., 2005; Wilhere, Goering & Wang, 2008). What is the adequate extent of representation of a feature in a reserve network? Williams (2001, p. 816) illustrates the definition of representation goal in the following way: ‘*A simple representation goal used to illustrate the basic principles in many academic studies has been to achieve at least one representation of every included species within the reserve network*. *Alternatively*, *the goal could be to achieve any required number of representations* (*this number could also differ among species*) *or could be expressed in terms of population size*, *probability of occurrence*, *etc*.’ Representation and efficiency become linked together in minimum set coverage planning (Pressey *et al*., 1997; Sarkar *et al*., 2006; Moilanen *et al*., 2009*c*; Cabeza *et al*., 2010), in which the goal is to satisfy all representation targets at minimum cost.

Interestingly, representation of features in reserves is not permanent and differs from the long-term persistence of those features in the landscape (Margules, Nicholls & Usher, 1994; Pressey & Logan, 1998; Knight *et al*., 2006*b*; Cabeza *et al*., 2010). This is because the distributions of species can change due to spatial (meta)population dynamics and population turnover: local populations may go extinct and empty sites become re-colonised. Among others, Williams (2001) pointed out that a clear distinction must be made between observation records of species and areas with high probability of long-term persistence.

Representation and representativeness sound closely related, have a similar history (Margules & Usher, 1981), are often discussed together, and are not always clearly distinguished from each other. Representation can be interpreted as the extent of occurrence of a particular species or other biodiversity feature within a specific area (Cabeza & Moilanen, 2001). Representativeness can be seen as a broader concept indicating whether a reserve network represents the full variety of biodiversity at all levels of organisation (Margules & Pressey, 2000). Representativeness may require long-term representation of many features across space.

### (6) Complementarity

Complementarity is often quoted as the defining concept of spatial conservation prioritisation within SCP, and in their review Margules & Pressey (2000, p. 249) define it as: ‘*All selection algorithms use complementarity*, *a measure of the extent to which an area*, *or set of areas*, *contributes unrepresented features to an existing area or set of areas*. *The precise measure depends on the targets that have been identified and on the type of data*. *Most simply*, *it can be thought of as the number of unrepresented species* (*or other biodiversity features*) *that a new area adds*.’

The historical development of the concept of complementarity is well known (Williams, 2001; Justus & Sarkar, 2002; Margules *et al*., 2002). Complementarity derives from studies of species composition and species richness. Whittaker (1972) related the notion of diversity to geographic scale and spatial context by introducing alpha, beta, and gamma diversity, which have enjoyed wide usage in ecology. Beta and gamma diversity together (=spatial turnover) were later recognised as underlying complementarity (Williams, 2001; Sarkar, 2006; Sarkar *et al*., 2006). Complementarity was first applied to SCP by Kirkpatrick, Brown & Moscal (1980) and was summarised by Kirkpatrick (1983, p. 128) in the context of maximising species richness in reserves in Tasmania: ‘*A major drawback of a listing of priority areas on the basis of a single application of a formula is that there is no guarantee that the priority area second or third on the list might not duplicate the species*, *communities or habitats that could successfully be preserved in the first priority area*. *In this paper I describe an iterative method that overcomes this difficulty and discuss its application to endemic species in Tasmania*.’ While the term complementarity was not used in that study, it clearly outlines a problem with scoring methods and describes an improved complementarity-based solution. Other early studies that developed implicitly complementarity-based ideas include Ackery & Vane-Wright (1984), Margules & Nicholls (1987) and Margules *et al*. (1988). Complementarity was apparently independently proposed by Rebelo & Siegfried (1990) when planning conservation efforts for Fynbos vegetation in South Africa.

The first mention of the term ‘complementarity’ was by Vane-Wright, Humphries & Williams (1991, p. 235) in their study comparing the biota of potential reserves: ‘*By employing complementarity*, *step-wise procedures can identify optimally efficient*, *single-site sequences of priority areas for a group*, *taking existing reserves into account or not*, *as required*.’ A major increase in publications related to this concept quickly followed with discussion about how exactly complementarity should be defined and applied (Belbin, 1993, 1995; Pressey *et al*., 1993; Colwell & Coddington, 1994; Pearce & Moran, 1994; Underhill, [Bibr b197]; Williams *et al*., 1996; Faith & Walker, 1996*a*; Csuti *et al*., 1997; Prendergast, Quinn & Lawton, 1999). Overall, it was accepted that complementarity had major advantages compared to, for example, using species richness scores as the basis for conservation (Margules & Pressey, 2000, p. 249): ‘*potential contribution of an area to a set of targets is dynamic – some or all of the features in an unselected area might have had their targets partly or fully met by the selection of other areas*. *In contrast*, *more traditional measures of conservation value such as species richness or the number of rare species are unresponsive to changing targets and decisions to reserve other areas*.’ Faith & Walker (1996*b*) and Faith *et al*. (2003) defined complementarity directly as ‘*the context-dependent*, *marginal gain in biodiversity provided by the area*’ (Faith *et al*., 2003, p. 311). Note that targets can also be specified, and complementarity evaluated in relation to various targets, including those specified for persistence of features (Faith & Walker, 1996*a*, *b*; Reyers, Van Jaarsveld & Kruger, 2000; Faith *et al*., 2003; Davis, Costello & Stoms, 2006; Williams *et al*., 2006). While complementarity is most commonly associated with computer-based site selection, Faith *et al*. (2003) consider it in a policy-based setting addressing the problem of implementing economic incentives and other instruments for biodiversity conservation.

Moilanen (2008) disagreed with the elevation of complementarity as a defining concept of SCP. He argued that there are optimisation methods that propose entire sets of sites as reserve networks, and the incremental definition of complementarity, the number of new targets covered, is irrelevant in the context of such methods. Complementarity is not a basic concept, but is in fact a property of a solution that arises from the requirements of comprehensiveness and efficiency as these two concepts require covering all features without duplication of effort. Furthermore, Moilanen (2008) argued that complementarity derives from the statistical concept of dependency: sites and conservation actions are not independent. Forms of dependence include that using resources for one site or species will detract from others. Second, conservation actions have variable effects on features; they may benefit some features while damaging others. Third, spatially close actions will have a joint effect *via* population-dynamical connectivity. Based on such considerations, Moilanen (2008, p. 1657) goes on to propose a generalised form of complementarity: ‘*conservation benefits of all conservation actions across the landscape should be evaluated jointly and account for long-term consequences of interactions between actions*. *This principle has explicit mathematical and technical meaning*, *and it implies that conservation benefits that follow from a particular conservation action at a site depend on the regional context of the site and conservation actions taken elsewhere*.’

### (7) Threat

Threat and another important SCP concept, vulnerability, are often tightly linked together without necessarily having a clear distinction. Below, we discuss these terms under the broad meaning that threat is about the presence of a process that may cause losses to biodiversity; vulnerability, on the other hand, is about the sensitivity of particular biodiversity features to a specific threat. Wilson *et al*. (2009, p. 24) describe the relevance of threats in the context of spatial prioritisation: ‘*The purpose of identifying priority areas for biodiversity conservation is to mitigate some of the processes that threaten biodiversity*. *Incorporating information on threatening processes and the relative vulnerability of planning units and features to these processes is therefore crucial for effective conservation prioritization*.’ Threat has long been recognised as a key factor in conservation biology. It has been threats to natural values that have led to the protection of many natural areas since the 1970s, broadly within the context of the ‘biodiversity crisis’ (Haila & Kouki, 1994; Sarkar *et al*., 2006). Margules & Usher (1981, p. 82) demanded more scientific criteria for selecting reserve sites: ‘*however*, *as a criterion the threat of human interference cannot be used on a site unless that site is highly valued on the criteria that are dependent upon scientific concepts*.’ The word ‘priority’ is used for the relative urgency of conservation action among different areas, and it is often determined from known threats which may change through time (Pressey, 1997; Pressey *et al*., 2000; Pressey & Taffs, 2001; Williams, 2001; Cowling *et al*., 2003). Threats are frequently accounted for in target-setting (Sarkar *et al*., 2006).

Threats were mentioned in the context of priority ranking in early reserve selection studies (Tans, 1974; Gehlback, 1975; Wright, 1977). Especially in older literature, threat has been compared to the concept of fragility which is often referred to as vulnerability (Ray, 1975; see Smith & Theberge, 1986, for review). In these early discussions, threat and vulnerability were frequently discussed together with diversity and rarity. Threats follow from the presence of threatening processes. This process perspective and the severity and imminence of threat are aspects that have been considered since the 1970s (Tans, 1974).

Classifications have been made also according to the spatial scale of threat (Reyers, 2004; Rodrigues *et al*., 2004*b*; Visconti *et al*., 2010*a*, *b*). Ultimate processes are the main causes of biodiversity loss that operate indirectly at global, national, or other broad scales. Instead, processes classified as proximate are dynamic physical expressions of ultimate processes, and they threaten biodiversity directly at regional or local scales (Lambin *et al*., 2001; Wilson *et al*., 2005*a*, *b*; Pressey *et al*., 2007). In addition, threats have been classified by their time scale of influence. Regan *et al*. (2007) define threats to be immediate if occurring within 5–10 years and Jarvis *et al*. (2010) reduced this to within 2–5 years. Long-term potential changes or future threats are also distinguished and expected in the 5–20 year time range (Jarvis *et al*., 2010). While one could argue about the time intervals considered as relevant, there is an important observation to be made here. In our interpretation, threat and vulnerability both have implications for the persistence of biodiversity into the future. Adequacy, comprehensiveness and representation can all be compromised due to threat and vulnerability.

Threats can originate from different societal, economic, demographic, technological, political, or cultural pressures (Lambin *et al*., 2001; Wilson *et al*., 2005*b*). One threat that has gained increasing attention is climate change (Araujo *et al*., 2004; Wilson *et al*., 2009; Rands *et al*., 2010), which is an ultimate threat in the sense that it cannot be stopped by local conservation action. Of course, even more fundamental ultimate threats of global population growth and increasing consumption underlie climate change. Proximate threats include logging, clearing, agricultural expansion, urbanization, grazing, expansion of infrastructure, mining, invasion by exotic species, hydrological changes, and salinization (Wilson *et al*., 2005*a*, *b*). Threats may be correlated to environmental factors such as climate, soil type, and topography, as well as geographic variables such as proximity to population centres and infrastructure, including roads and irrigation systems. Pressey *et al*. (2007) emphasised that proximate threats are dynamic and they require planners to track and predict spatial changes in threats over time.

Threat can also be analysed at the species level, influencing allocation of conservation effort among species (Bonn, Rodrigues & Gaston, 2002). Perhaps the most well-known system to determine the threat status of a species is the IUCN Red List, which applies criteria primarily related to range size and population trends (Gärdenfors *et al*., 2001; IUCN, 2001, 2010; Redding & Mooers, 2006). The red list status has often been considered as a synonym of the conservation priority of a species (Possingham *et al*., 2002), and conservation organisations have formulated their strategies around these priorities (Myers *et al*., 2000; Brooks *et al*., 2006; Jarvis *et al*., 2010).

### (8) Vulnerability

Vulnerability is complementary to threat, and can be defined as the risk of the area being transformed *via* damage caused to biodiversity features by threatening processes. Before the emergence of the specific term vulnerability, the concept was discussed as fragility. Fragility has been recognised as a high probability of extinction or damage of a species, feature, or system and conservation or protection has been seen as a natural consequence for those fragile features (Ratcliffe, 1977; Wright, 1977; see Smith & Theberge, 1986, for review). Fragility was still in use at the beginning of the 1990s, and was defined as the inverse of ecosystem stability (Nilsson & Grelsson, 1995). The definition of Pressey, Johnson & Wilson (1994), Pressey, Possingham & Margules (1996) and Pressey *et al*. (1996) can be recognised as the first definition of term ‘vulnerability’ in SCP. Pressey *et al*. (1996, p. 328) state: ‘… *the development of priorities should recognise that environmental units or other land classes vary in their vulnerability to threatening processes and*, *therefore*, *in their need for protection*.’ Vulnerability is a measure of the likelihood of the biodiversity in an area being lost to current or threatening processes. Vulnerable areas are most likely to be lost and their loss will have the most serious impact on the achievement of targets (Margules & Pressey, 2000; Pressey, Cowling & Rouget, 2003; Margules & Sarkar, 2007). Vulnerability is a concept also used in the literature of risk and hazard (see Cutter, 1996, for review).

Vulnerability has been used to define hotspots, which Myers (1988, 1990) and Myers *et al*. (2000) defined as localities with exceptional concentrations of species and levels of endemism, and exceptional degrees of threat. Species endemism has been proposed as a simple measure of vulnerability (Brooks *et al*., 2006) but the relationship between endemism and vulnerability also has been criticised (Orme *et al*., 2005). Following Faith & Walker (1996*b*, p. 418): ‘*An area that would contribute many so-far unrepresented species to an existing reserve system is arguably a particularly high priority for protection efforts if it is also rated as vulnerable*.’ It has been proposed that areas could be prioritised according to both representation and vulnerability of sites (Pressey, 1997; Pressey *et al*., 2000; Pressey & Taffs, 2001; Cowling *et al*., 2003; Lawler, White & Master, 2003). The more commonly accepted approach is presently prioritisation by combination of vulnerability and irreplaceability (Gaston, Pressey & Margules, 2002; Noss *et al*., 2002; Pressey, Watts & Barrett, 2004; Brooks *et al*., 2006; Section III.11). While vulnerability itself is easy to understand, it is not fully clear how it should drive conservation priority and decisions. Usually the interpretation is that high vulnerability implies high priority, but one can also argue that areas of high vulnerability should be avoided so as to avoid compromising successful conservation (Game *et al*., 2008). Threat and vulnerability are closely linked to the topic of scheduling of conservation action and sequential reserve selection. In scheduling, a temporal dimension is added to prioritisation, and the question concerns what order of conservation action will produce the most beneficial long-term conservation outcome given that habitats and sites are being degraded at rates that vary depending on the habitat type, location and ownership (Pressey *et al*., 2004; Strange, Thorsen & Bladt, 2006; Wilson *et al*., 2007; Knight *et al*., 2010). Accounting for human and social factors allows for more efficient scheduling of conservation actions (Knight *et al*., 2010).

Pressey & Taffs (2001, p. 372) emphasised the importance of environmental features: ‘*Vulnerability is likely to be linked partly to geography* (*e*.*g*. *distance to timber mills*) *but also to the environmental attributes of areas* (*e*.*g*. *ruggedness*, *surface geology*) *that help to determine species composition*. *Areas in similar parts of the ordination space with similar species will therefore often have similar vulnerabilities*.’ According to Wilson *et al*. (2005*b*), conservation planning still lacked a consistent definition of vulnerability. They proposed a new definition (Wilson *et al*., 2005*b*, p. 529) that characterises vulnerability in three components: ‘*Two of our dimensions of vulnerability – exposure and intensity – apply to areas and consequently to the features they contain*. *The third dimension – impact – applies only to features*. *However*, *this shift in focus from areas to features does not apply in all cases*.’ Here, exposure is the probability of a threatening process affecting an area over a given time period, or alternatively, the expected time until an area is affected. Intensity refers to the strength of the threatening process (magnitude, frequency, and duration), and impact reflects the response features to the threat (Wilson *et al*., 2005*b*, 2009). We see potential for confounding threat and vulnerability in the definitions above, and we return to these differences in Section IV.

### (9) Efficiency

The concept of efficiency stems from the fact that the area of land (or money) available for conservation is limited (Margules & Pressey, 2000; Williams, 2001). The appearance of efficiency in SCP can be traced to the debate between scoring *versus* iterative complementarity-based approaches in reserve selection. The first definition of efficiency appeared in the context of scoring by Pressey & Nicholls (1989*b*, p. 200–201): ‘*This paper defines a measure of the effectiveness of procedures for conservation evaluation in promoting the basic conservation goal*. *This measure is referred to as* “*efficiency*” *and its values are calculated for a variety of scoring criteria using two large data sets*. *Efficiency of sampling is defined here by the following formula*: *E* = *1* – (*X*/*T*) *where E is efficiency*, *X is the number or extent of highest ranking sites needed to contain all attributes a given number of times*, *and T is the total number of area of sites*.’ By this definition, efficiency is high when only a small number of sites are needed to cover required attributes. Pressey & Nicholls' (1989*a*, *b*) publications initiated an expanding discussion on possibilities of site-selection algorithms and conservation efficiency. Efficiency, as a term, has a multitude of definitions in economics and engineering. Efficiency in the context of spatial prioritisation frequently implies cost efficiency, which can be defined as the ratio of benefits to costs (Pearce & Moran, 1994; Ando *et al*., 1998; Juutinen & Mönkkönen, 2004; Naidoo *et al*., 2006; Wilson *et al*., 2009). Efficiency is also called economy in parts of the literature (Sarkar *et al*., 2006; Margules & Sarkar, 2007). The use of optimisation to find efficient protected area solutions is motivated by a belief that such solutions have relatively higher social acceptability and therefore stand a greater chance of being implemented (e.g. Fernandes *et al*., 2005; Game *et al*., 2011).

Early on, conservation planners recognised that most regions have alternative possible configurations for CAR protected area networks, and that the spatial extent of a protected area network influences its cost, thereby suggesting a focus on efficiency (JANIS, 1997). Later, efficiency found its way deeply into the complementarity and targets-based planning paradigm (Williams, 2001). Site-selection algorithms, which use complementarity, can identify areas that meet biodiversity targets as efficiently as possible (Bedward *et al*., 1992; Pressey *et al*., 1994; Csuti *et al*., 1997). The minimum set coverage approach to site selection represents all features with minimal cost, and the approach therefore is explicitly focusing on (cost-) efficiency (Van Jaarsveld, 1995; Williams, 2001; Pressey *et al*., 2004; Moilanen *et al*., 2009*c*). In another analysis variant, the maximal coverage problem, conservation targets for all features cannot be met within constraints. In this case, efficiency is higher when more features have targets met within the maximum number, total extent or total cost of conservation areas (Camm *et al*., 1996; Csuti *et al*., 1997; Pressey *et al*., 1997; Cameron, Williams & Mitchell, 2008). Cost is frequently related to the area of land or sea protected, and may include purchase and management costs, or the costs of lost economic development (Juutinen & Mönkkönen, 2004; Possingham *et al*., 2006; Margules & Sarkar, 2007). Efficiency also has weaknesses: it implies no loss or reduction of feature representation before (or after) conservation is implemented. This assumption may be invalid in many situations (Pressey *et al*., 2004).

Later, Possingham *et al*. (2006, p. 520) emphasises the advantages of applying efficiency in reserve design: ‘*Efficiency is important*, *because it minimizes the possibility of constructing a reserve system that is too large and expensive to manage*. *An efficient reserve system is more likely to succeed in the face of competing interests*.’ Efficiency has also been called an established measure of effectiveness (Van Jaarsveld, 1995; Margules & Pressey, 2000; Pressey *et al*., 2004; Section III.10). For instance, Knight *et al*. (2010) minimised the number of private land managers involved with a candidate reserve network, thereby also improving the effectiveness of implementation *via* efficiency.

### (10) Effectiveness

Effectiveness is a more holistic concept than efficiency. Rodrigues *et al*. (1999, p. 1453) defined it early as: ‘*effectiveness is measured as the gap between the representation target required and the one attained by the existing network*.’ Conservation can be said to be effective when representation of all ecosystems and species (biodiversity) is adequately fulfilled (Gaston *et al*., 2006). Effectiveness and efficiency are somewhat closely related concepts that are often mentioned together. Margules & Pressey (2000, p. 243) describe effectiveness as follows: ‘*The effectiveness of systematic conservation planning comes from its efficiency in using limited resources to achieve conservation goals*, *its defensibility and flexibility in the face of competing land uses*, *and its accountability in allowing decisions to be critically reviewed*.’ We note that this definition pertains to the effectiveness of spatial conservation prioritisation. A narrower way to define effectiveness is meeting all specified protection or representation targets for a region (Rodrigues & Brooks, 2007; Wiersma & Nudds, 2009; Ferrier & Drielsma, 2010). Ferrier & Wintle (2009, p. 12) describe the concept as: ‘*Measures based on such targets* (*e*.*g*. *number of targets achieved*, *or average proportion of each target achieved*) *step beyond simply evaluating representativeness to also consider adequacy thereby assessing what we here call the* “*conservation effectiveness*” *of a reserve network*.’ The ideas underlying effectiveness developed through considerations of representation and representativeness across different spatial scales, and through different levels of hierarchical organisation of biodiversity (Noss, 1990; Nilsson & Götmark, 1992; Yahnke, de Fox & Colman, 1998; Pressey & Taffs, 2001; Pressey *et al*., 2002).

Addressing the effectiveness of conservation is a challenge (Meir *et al*., 2004; Wilson *et al*., 2007). The two most obvious and basic measures proposed for effectiveness, the number and extent of areas, have been found to be insufficient criteria (Pressey & Taffs, 2001; Downsborough, Shackleton & Knight, 2011). Additional considerations include those of spatial scale, connectivity, and features of reserve areas (Saunders *et al*., 1991; Rebelo & Siegfried, 1992; Pressey & Taffs, 2001; Rodrigues *et al*., 2004*b*; Gaston *et al*., 2006; Cantu-Salazar & Gaston, 2010). Also measures of feature distribution, condition, persistence and viability could be employed to link effectiveness to adequacy (Margules & Sarkar, 2007). Gaston *et al*. (2006) summarised that the objective measurement of ecological effectiveness should: (*i*) enable more robust claims of conservation successes; (*ii*) provide opportunities to learn from and respond to conservation successes, failures, or inadequacies; (*iii*) improve the efficiency and effectiveness of conservation action, including future site designation; (*iv*) make it more difficult for development processes to challenge protected area designations and any associated restrictions; (*v*) facilitate appropriate, targeted management action at both the local and national level; and (*vi*) reduce the potential for skepticism among policymakers, funding agencies, land owners, and others of the long-term value of conservation efforts.

Increased awareness about the importance of socio-political factors in the implementation and success of conservation has introduced new perspectives into conservation effectiveness. Interest in the measurement and verification of conservation impact has grown, and effectiveness has been expanded to cover factors such as management, governance, and costs (Wright *et al*., 2007; Brooks, Wright & Sheil, 2009; Eklund *et al*., 2011). The concept of management effectiveness (how well conservation areas are managed) has also evolved from the concept of effectiveness (Chape *et al*., 2005; Hockings *et al*., 2006; Cantu-Salazar & Gaston, 2010). Knight *et al*. (2010) describes in detail human and social factors which influence the effectiveness of translating maps of priorities or opportunities into action. As Knight *et al*. (2011*a*) emphasise, human, social, and financial capital can be seen as major determinants of the effectiveness of implemented conservation action. Conservation effectiveness is a concept that is still evolving. We shall clarify the (somewhat unclear) relationship between adequacy, comprehensiveness, representativeness and effectiveness in Section IV.

### (11) Irreplaceability

Irreplaceability is a concept almost exclusively used in the context of conservation biology. Heuristically, irreplaceability reflects how important a specific area is for the efficient achievement of conservation objectives (Pressey *et al*., 1993, 1994; Ferrier, Pressey & Barrett, 2000; Margules & Pressey, 2000; Williams, 2001; Possingham *et al*., 2006; Carwardine *et al*., 2007). Irreplaceability is related to the existence of alternatives in reserve design, and provides a quantitative assessment of the contribution of areas for meeting conservation targets (Pressey *et al*., 1993, 1994; Ferrier *et al*., 2000; Justus & Sarkar, 2002). A completely irreplaceable area is considered essential for meeting conservation objectives, whereas an area with low irreplaceability can be substituted by other sites. In older conservation literature a precursor of irreplaceability was replaceability (Smith & Theberge, 1986, p. 729): ‘*Replaceability or the availability of similar alternative sites is sometimes assessed*…’ Irreplaceability was first applied to conservation planning in New South Wales, and after that to the selection of priority areas in South Africa and USA (Pressey *et al*., 1993, 1994; Rebelo, 1994; Lombard *et al*., 1997). Most scientific discussion on irreplaceability took place during the decade after the first introduction of the concept.

The first definition of irreplaceability was given by Pressey *et al*. (1993) and Pressey *et al*. (1994). Irreplaceability can be viewed in two contexts, as Pressey *et al*. (1994, p. 243) put it: ‘*The frequency of occurrence in individual sites in the range of possible representative systems then provides a novel and useful index of the importance of each site to conservation*’. *This index is referred to here as* ‘*irreplaceability*’ *which can be defined in two ways*: (*i*) *Irreplaceability is the potential contribution of any site to a reservation goal*; *and* (*ii*) *Irreplaceability is the extent to which the options for a representative reserve system are lost if that site is lost*.' Pressey *et al*. (1993, p. 126) commented: ‘*Irreplaceability therefore provides a fundamental way of measuring the conservation value of any site*.’ Belbin (1995) suggested that irreplaceability of any site could be defined as the distance in multivariate space to its nearest neighbour and reflects the degree of isolation from the closest neighbour as an estimate of ecological distinctiveness. Rebelo (1994) defined global irreplaceable areas as areas with unique species contrasting the term with conditional irreplaceability. Therefore, irreplaceability has been referred to also as a measure of uniqueness, i.e. as the relative importance of a site (Funk & Richardson, 2002; Lawler *et al*., 2003).

Calculation of irreplaceability considers complementarity in an implicit manner (Pressey & Taffs, 2001; Lawler *et al*., 2003), and as a practical limitation, it can, in its original form, only be measured exactly for small datasets (Pressey *et al*., 1994; Carwardine *et al*., 2007). Early developments of irreplaceability aiming at improving its applicability include work by Ferrier *et al*. (2000), who introduced a new statistical approach to the estimation of irreplaceability together with a comprehensive review of the prior usages of the term. Since then, several different operational definitions of irreplaceability have been used. The original definition of irreplaceability has been called exact irreplaceability (Pressey *et al*., 1993; Wilson *et al*., 2009). The statistical estimator of Ferrier *et al*. (2000) has been called estimated irreplaceability (Wilson *et al*., 2009). Further, so-called summed irreplaceability has been measured when there are targets for many features to achieve (Ferrier *et al*., 2000).

Computation of irreplaceability has also been discussed in the context of reserve-selection optimisation algorithms (Noss *et al*., 2002; Stewart *et al*., 2003). Wilhere *et al*. (2008) see optimisation algorithms as the wrong tool for estimating irreplaceability as there is no guarantee that any optimisation method explores the solution space in the unbiased manner required for a conceptually correct calculation of irreplaceability. Sometimes, irreplaceability is estimated from multiple outputs produced by stochastic optimisation (Ball, Possingham & Watts, 2009). In this case the measure of irreplaceability is actually selection frequency, arising as a combination of site importance and stochastic convergence (or lack of it) of optimisation.

Pressey *et al*. (1993) linked vulnerability to irreplaceability. Combining these two concepts has been widely considered a feasible approach to conservation prioritisation – sites that need conservation action urgently are vulnerable and highly irreplaceable (Pressey & Taffs, 2001; Pressey *et al*., 2004; Linke *et al*., 2007).

### (12) Replacement cost

Replacement cost is a more recent spatial prioritisation concept developed by Cabeza & Moilanen (2006). It is related to, but has a significantly different operational definition from irreplaceability (Pressey *et al*., 1993, 1994; Section III.11). While irreplaceability indicates the likelihood of needing a site for achieving a specific conservation target, replacement cost measures the cost by which we can forcibly exclude a group of sites from conservation or forcibly include a group of sites into a reserve network. Cabeza & Moilanen (2006, p. 336) define replacement cost in the following way: ‘*Replacement cost refers to the loss in solution value given that the optimal cost-efficient solution cannot be protected and alternative solutions*, *with particular sites forcibly included or excluded*, *are needed*. *This cost can be defined either in terms of loss of biological value or in terms of extra economic cost*’. The concept of replacement cost can be divided into inclusion cost and exclusion cost. Heuristically, inclusion cost is the loss following forced inclusion of poor-quality sites into a conservation solution. Exclusion cost is the reduction in solution quality that must be accepted when high-quality sites must remain outside conservation. As one specific detail, calculation of replacement cost requires full re-optimisation for the constrained solution: the constrained solution is not simply the optimal solution minus the areas that cannot be had (Cabeza & Moilanen, 2006).

Some authors have seen replacement cost as a theoretical approach which can work with any quantitative measure of conservation effectiveness, including predictions of biodiversity persistence (Ferrier & Drielsma, 2010). In a general form, replacement cost can be seen as the difference in value between an optimal unconstrained solution and another solution, which has been developed under some (arbitrary) constraints (Moilanen *et al*., 2009*a*; Moilanen, Kujala & Leathwick, 2009*b*). As irreplaceability, replacement cost is fundamentally dependent on the availability of alternative conservation solutions (Moilanen *et al*., 2009*a*). While replacement cost is best defined in the context of the benefit function approach to conservation (Arponen *et al*., 2005), it can also be applied in the context of target-based planning. In this case replacement cost is simply the number of targets that are failed due to constraints on the solution, or, the extra cost that must be paid to satisfy targets.

As a relatively recent concept, and one founded in a mathematic definition, alternative definitions for replacement cost have not been proposed. It is the favoured method for evaluating the importance of individual areas or groups of areas using the Zonation software (see Moilanen *et al*., 2009*b*, 2011, for references).

### (13) Flexibility

The concept of flexibility was formulated in the early 1990s together with the complementarity-based planning paradigm (Pressey *et al*., 1993, 1994; Van Jaarsveld, 1995; Church, Stoms & Davis, 1996; Williams *et al*., 1996). According to the early definition of flexibility by Pressey *et al*. (1993, p. 125): ‘*The more alternative networks that can be appraised*, *the more likely the planner is to find one which is not only representative but also maximizes values of design and land suitability and*/*or minimizes costs*.’ Rodrigues, Cerdeira & Gaston (2000, p. 565) stressed that: ‘*Flexibility can be addressed a priori*, *when devising the problem to be solved*, – *or a posteriori*, *by modifying the reserve network obtained by a selection procedure*.’ Therefore, flexibility is a concept which refers to opportunities to choose and use alternatives in SCP.

More recently, flexibility has been seen as a significant advantage in SCP. It is useful if the conservation goals can be met in different ways, increasing the likelihood of successful implementation of conservation (Stewart *et al*., 2003; Araujo *et al*., 2004; Possingham *et al*., 2006; Wilson *et al*., 2009). According to Moilanen (2008), flexibility needs to be offered to stakeholders so that goals that have not been specified in quantitative numeric form can be satisfied. Knight *et al*. (2011*b*) found that flexibility manifests both as (*i*) the range of alternative site configurations that would achieve targets to form a representative protected area network, and (*ii*) the ability to respond to opportunities dynamically as they arise (i.e. informed opportunism). This latter principle of informed opportunism is an important recent addition to the scope of flexibility as it can be seen that flexibility enables effectiveness (Game *et al*., 2011; Knight *et al*., 2011*a*).

Flexibility may have linkages to irreplaceability (Ferrier *et al*., 2000). Following the definition of Margules *et al*. (2002, p. 321): ‘*Flexibility is a property of the network of areas*. *It arises because many of the areas needed to fulfil the representation goal can be replaced by one or more others*. *Flexibility refers to the different spatial arrangements of areas available to achieve the goal*.’ Williams (2001, p. 813) highlights it as: ‘*The degree to which alternatives exist for one or more selected areas in the context of reaching a particular conservation goal*.’ In both these definitions a linkage to irreplaceability is implicit: if an area has high irreplaceability, there cannot be much flexibility, and *vice versa*. Williams (2001) categorised areas according to their flexibility as absent, incomplete (replacing the selected area while still reaching the conservation goal would require substitution of two or more areas or one or more areas of greater cost), or complete (other areas could be substituted, one-for-one by number or by cost, with the current choice). We point out that these site-specific considerations of flexibility actually closely resemble the definition of irreplaceability (Section III.11), and also are at odds with the later definitions of Pressey *et al*. (2003, 2004), who treated flexibility as a property of a reserve network or solution, not as a property of an individual area.

The operational mechanics of flexibility are a different matter. The implication of flexibility is that one would need multiple spatially different solutions (allocations of conservation action) that all nevertheless are close to optimal in terms of the conservation value of the solution. As one example, the Marxan software (Ball & Possingham, 2000) can produce alternative solutions: the stochastic optimisation (simulated annealing) employed by Marxan will naturally produce slightly different solutions in different optimisation runs. Alternatives can be picked from amongst these.

## IV. CRITICISMS AND CLARIFICATIONS OF THE CONCEPTS

**Fig. 3:**
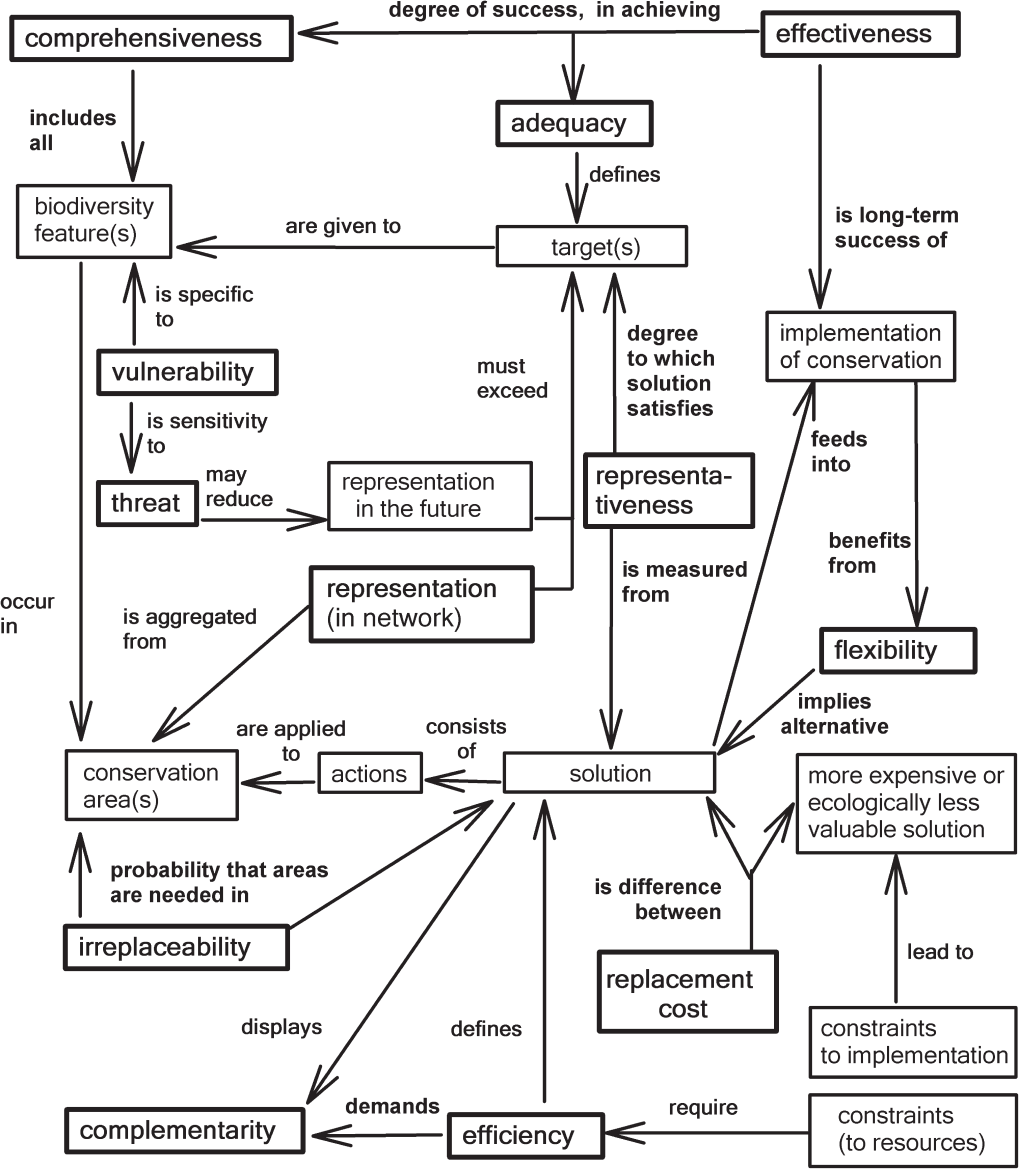
A concept diagram for the core concepts of systematic conservation planning, which are shown in bold font in boxes. Supplementary concepts are shown in a plain font. Arrows link the concepts, and the type of linkage is read in the direction of the arrow, e.g. ‘vulnerability is sensitivity to threat’.

There are thousands of publications that apply some of the concepts of SCP (Table 1). Hundreds of studies discuss individual concepts and their definition or application. Thus, it is no wonder that interpretations of the concepts may vary, and conflicting usages occur. Here we return to all 12 terms together and attempt to clarify their meaning and differences, concentrating on issues we subjectively find potentially confusing. We point out that for a set of definitions to make sense, they must make sense together, without conflicts between terms and without different terms meaning the same thing (Regan, Colyvan & Burgman, 2002). Concepts should also correspond to generally accepted usage of the English language. Ideally, the set of concepts utilised in a conceptual model should be sufficient, complete and parsimonious (Novak & Gowin, 1984; Wallace & Mintzes, 1990). We approach the core concepts of SCP from this perspective. Figure 3 shows one way of linking the core SCP concepts using a concept map.

We start from the CAR terms: comprehensiveness, adequacy, and representativeness (Commonwealth of Australia, 1992; Sections III.2–4). The interpretation of these terms in our opinion is clear if comprehensiveness and adequacy are taken as properties of the objective and representativeness as a property of the solution (Fig. 3). Comprehensiveness is about covering the full spectrum of biodiversity, i.e. the set of features that sufficiently measure biodiversity in the broad sense (Possingham *et al*., 2006), according to the aims of the study. Adequacy requires that high enough targets should be given to features to ensure that the conservation outcome can be taken as favourable (Pressey *et al*., 2000; Williams & Araujo, 2000; Klein *et al*., 2009). Representativeness (Margules & Pressey, 2000; Ferrier & Wintle, 2009), on the other hand, can be seen as a property of a reserve network, or conservation solution in a broader sense. We see representativeness as the degree to which the reserve network satisfies the requirements of comprehensiveness and adequacy. These definitions generally agree with published literature, although the difference between comprehensiveness and representativeness as properties of objectives and the solution, respectively, is not generally clearly stated (Possingham *et al*., 2005, 2006; Crossman & Bryan, 2006).

Note that it is possible for a reserve network to be representative without being comprehensive, or, that it can be comprehensive without being representative. The former occurs when features in analysis include only a small subset of the biodiversity features in the region (e.g. only birds), but the network is fully representative for this subset of features. The latter occurs when features cover the full spectrum of biodiversity, but data are available for, or the reserve network only covers, part of the features. This would be the case, for example, if good data were available for mammals, birds, and amphibians, but the true comprehensive conservation objective requires conservation across all higher taxa. Finally, note that recent definitions may differ subtly from early definitions. For example, according to our interpretation above, the definition of comprehensiveness in Wilson *et al*. (2009) combines elements of both comprehensiveness and representativeness.

Representation is a fundamental term that is close to the operational biogeographical reality of SCP (Section III.5; Pressey & Logan, 1998; Margules & Pressey, 2000; Williams, 2001). In our view, and agreeing with literature, representation is about the occurrence level of features in a specific area. This area could be one site, or a group of sites (reserve network). The occurrence level could be measured, e.g. in terms of abundance, density, probability of occurrence, or habitat coverage. The specific measurement is relevant operationally.

Efficiency (Section III.9; Pressey & Nicholls, 1989*a*, *b*) is another rather clear concept, with the general idea having analogies to engineering and economics: high yield per unit investment or high output per input unit. In the context of SCP, efficiency would most commonly be cost efficiency, that is, benefits per unit costs (Ando *et al*., 1998; Margules & Sarkar, 2007; Wilson *et al*., 2009). When conservation targets are satisfied with minimum cost in the minimum set coverage context, cost efficiency becomes maximised implicitly. We view efficiency as a quantity that should be measurable from a solution, based on information about target achievement and costs.

While a clear distinction is not always apparent, effectiveness (Section III.10) has to mean something different from efficiency if duplication of meaning is to be avoided. Our interpretation of the sense of the literature (e.g. Gaston *et al*., 2006; Ferrier & Wintle, 2009) is that effectiveness is a more holistic and inclusive concept than efficiency. Effectiveness is defined with respect to goals. It must answer the needs of comprehensiveness and adequacy, and implies solutions that can be successfully implemented and maintained into the future. If persistence is among the conservation goals, additional criteria such as the likelihood of vegetation change must be considered (Pressey & Taffs, 2001). Effectiveness can perhaps also include criteria that cannot be implemented in a quantitative form in site selection or spatial prioritisation (Chape *et al*., 2005; Knight *et al*., 2011*a*). If effectiveness is defined in this manner, one could even argue that effectiveness does not necessarily imply efficiency, except for the fact that cost-efficient solutions may be generally easier to implement than expensive solutions (Possingham *et al*., 2006). But, an expensive solution could be effective as well.

Threat and vulnerability (Sections III.7, 8) is a pair of concepts that can be split into several subcomponents (Wilson *et al*., 2005*b*; Visconti *et al*., 2010*a*, *b*), and which may be confounded in the literature. In our opinion, the following way of organising the concepts is logically consistent and corresponds to the general sense of the literature to date. First, threat and vulnerability are concerned with the persistence of biodiversity into the future (Margules & Pressey, 2000). They can be specific to individual features in individual areas as different features may be impacted by different threats (Pressey *et al*., 2007). For threat and vulnerability to have operational relevance, there must first be representation of some feature(s) in the area. If there is no representation, there is nothing to lose. Second, we would define vulnerability of the expected degree of loss of representation for a feature, conditional to the intensity of some specific threat. Third, threat defines the presence or intensity of the specific threat in the area. Summarising, representation defines what there is to lose, vulnerability defines expected loss conditional on the presence of the threat, and threat defines the intensity by which the threat is present. Thus, representation, vulnerability and threat become linked, but they all have completely non-overlapping definitions (Fig. 3).

Irreplaceability and replacement cost (Sections III.11, 12; Pressey *et al*., 1993, 1994; Ferrier *et al*., 2000; Cabeza & Moilanen, 2006) are operational measures that, heuristically expressed, aim to express how important an area is for conservation. Is it critically important or could it be replaced by other alternatives with little or no loss in target achievement or efficiency? Irreplaceability has several slightly different operational definitions and it is sometimes confounded by selection frequency or alternative solutions produced by stochastic optimisation (Ball *et al*., 2009). Replacement cost has a mathematically clear definition (Cabeza & Moilanen, 2006), measured *via* the difference between an optimal unconstrained and optimal constrained solution. In these definitions, area could be a single site or set of sites. We point out that both irreplaceability and replacement cost could be defined in terms of conservation actions in general, not only in terms of reserve selection.

Flexibility (Section III.13; Pressey *et al*., 1993, 1994) is another concept that is not altogether clearly defined in the SCP literature. It implies the ability to achieve targets in different ways, at the level of the network or conservation solution (Margules *et al*., 2002; Wilson *et al*., 2009). Flexibility differs from irreplaceability in that it would not be measured for a specific area. A flexible solution could perhaps be easily modified to answer changed needs of alternative land uses (Fig. 3). Flexible solutions might answer objectives that stakeholders have not made public. In our interpretation, flexibility in SCP implies that there are alternative solutions to the targeting of conservation action. Alternatives can be taken advantage of in negotiations relevant to land-use planning, and in that sense flexibility may beget effectiveness.

Complementarity (Section III.6) is the concept that is said to define the field of SCP most clearly (Margules & Pressey, 2000; Williams, 2001). While the original definition of complementarity (Vane-Wright *et al*., 1991), i.e. the number of targets covered by addition of sites, is clear, this definition does not stand scrutiny. For example, this definition ceases to exist when the solution is generated by an optimisation method that proposes sets of sites and does not construct the solution by iterative additive selection of individual areas (Moilanen, 2008). A fundamental concept and its definition cannot be conditional to the use of one particular type of optimisation algorithm (Underhill, [Bibr b197]). Rather, we prefer a generalised definition of complementarity, which states that the concept is effectively about how conservation actions work synergistically together, to achieve the objectives of comprehensiveness and adequacy in an efficient manner (Moilanen, 2008). Complementarity is a property of an efficient and comprehensive solution – actions must work together efficiently or else the solution will be somehow ecologically suboptimal or economically inefficient.

## V. CONCLUSIONS

1. Systematic conservation planning (SCP) is a discipline that influences land-use decisions at the global scale. Our review follows the development of SCP through 12 key concepts while showing differences between terms in their history and clarity of meaning. We concentrated on those terms relevant for goal-setting and problem solution applied in spatial prioritisation within the biogeographical component of SCP. We highlight the importance of discussion about terminology. Conservation biology has a global scope, with vastly different stakeholders involved including environmental managers, scientists, private interests, national administrations, research institutes, international NGOs, land owners, small businesses, international conglomerates, etc. With people from such varied backgrounds involved, it is vital that stakeholders understand each other (Prendergast *et al*., 1999; Knight *et al*., 2006*b*, 2011*a*).
2. Older concepts and theories were the foundation upon which newer SCP concepts such as complementarity, irreplaceability, and efficiency were built. Before SCP was developed, conservation biology focused on concepts such as rarity, richness, diversity, patch size, and naturalness (Goldsmith, 1975; Wright, 1977; Margules & Usher, 1981; Soulé, 1985; Smith & Theberge, 1986; Usher; 1986), and all were used as criteria for defining an ideal reserve. Threat was also recognised as part of conservation biology at that time (Tans, 1974; Gehlback, 1975; Ray, 1975; Ratcliffe, 1977; Smith & Theberge, 1986). We find that these concepts have both direct and indirect connections to basic ecological theories such as island biogeography theory, species-area relationships, the ‘single large or several small’ debate, species distribution patterns in naturally fragmented habitat, and metapopulation dynamics (MacArthur & Wilson, 1963; Diamond, 1975; Connor & McCoy, 1979; Saunders *et al*., 1991; Rebelo & Siegfried, 1992; Justus & Sarkar, 2002; McCarthy *et al*., 2011).
3. We define the key question of spatial conservation prioritisation inside SCP as follows: how to create a system of protected areas that conserves as much of a region's biodiversity, species, and habitats (representation) while covering different spatial scales and compositional and hierarchical levels (comprehensiveness, representativeness) and also taking these principles into account in the long term (adequacy and persistence). In the real world, not all biodiversity and ecosystems can be protected and consequently we must apply the complementarity principle and economic objectives (efficiency, cost effectiveness) so that we can achieve effective solutions. We should ensure that critical biodiversity is adequately protected (irreplaceability, replacement cost), but in a flexible (flexibility) manner that allows for implementation and integration with the future needs of competing land uses (vulnerability, threat). Systematic conservation planning (SCP) then includes spatial prioritisation as a data-driven component of a social collaboration process that aims at enabling and facilitating implementation of conservation action.
4. From our review it is apparent that the core terms of SCP have had variable definitions and they have been used assuming slightly different meanings. Such variation, undesirably, introduces linguistic uncertainty (Regan *et al*., 2002; Bonn & Gaston, 2005; Carey & Burgman, 2008; Regan, Ensbey & Burgman, 2009) into discussions, negotiations, and planning. Many of the publications reviewed herein have used the core SCP terms without being explicit about which alternative definition is actually employed. Few publications have focused on defining concepts or terminology (e.g. Pressey *et al*., 1993; Justus & Sarkar, 2002). This review clarifies the history and meaning of these core terms of SCP.
5. New understanding and new challenges to conservation biology might yet necessitate further development of the core set of SCP concepts. There remain further questions about these concepts and their interrelations. Is this set of concepts parsimonious? Would further concepts be helpful? Are the definitions of the concepts sensible from the perspective of semantic analysis? Are the operational definitions of these concepts clear and applicable in real-world planning? Also, the present work has not considered analysis of the major socio-political component of SCP, which has its own suite of concepts that have been discussed elsewhere (Knight *et al*., 2006*a*, 2010; Margules & Sarkar, 2007; Pressey & Bottrill, 2009).
